# Development of a novel prediction model for differential diagnosis between spinal myxopapillary ependymoma and schwannoma

**DOI:** 10.1038/s41598-023-50806-w

**Published:** 2024-01-02

**Authors:** Chorog Song, Hyun Su Kim, Ji Hyun Lee, Young Cheol Yoon, Sungjoon Lee, Sun-Ho Lee, Eun-Sang Kim

**Affiliations:** 1 Department of Radiology, Samsung Medical Center, Sungkyunkwan University School of Medicine, 81 Irwon-Ro, Gangnam-Gu, Seoul, 06351 Korea; 2 Department of Neurosurgery, Samsung Medical Center, Sungkyunkwan University School of Medicine, Seoul, Korea

**Keywords:** Diagnostic markers, Cancer imaging, Oncology, Tumour biomarkers, Nervous system

## Abstract

Spinal myxopapillary ependymoma (MPE) and schwannoma represent clinically distinct intradural extramedullary tumors, albeit with shared and overlapping magnetic resonance imaging (MRI) characteristics. We aimed to identify significant MRI features that can differentiate between MPE and schwannoma and develop a novel prediction model using these features. In this study, 77 patients with MPE (*n* = 24) or schwannoma (*n* = 53) who underwent preoperative MRI and surgical removal between January 2012 and December 2022 were included. MRI features, including intratumoral T2 dark signals, subarachnoid hemorrhage (SAH), leptomeningeal seeding, and enhancement patterns, were analyzed. Logistic regression analysis was conducted to distinguish between MPE and schwannomas based on MRI parameters, and a prediction model was developed using significant MRI parameters. The model was validated internally using a stratified tenfold cross-validation. The area under the curve (AUC) was calculated based on the receiver operating characteristic curve analysis. MPEs had a significantly larger mean size (*p* = 0.0035), higher frequency of intratumoral T2 dark signals (*p* = 0.0021), associated SAH (*p* = 0.0377), and leptomeningeal seeding (*p* = 0.0377). Focal and diffuse heterogeneous enhancement patterns were significantly more common in MPEs (*p* = 0.0049 and 0.0038, respectively). Multivariable analyses showed that intratumoral T2 dark signal (*p* = 0.0439) and focal (*p* = 0.0029) and diffuse enhancement patterns (*p* = 0.0398) were independent factors. The prediction model showed an AUC of 0.9204 (95% CI 0.8532–0.9876) and the average AUC for internal validation was 0.9210 (95% CI 0.9160–0.9270). MRI provides useful data for differentiating spinal MPEs from schwannomas. The prediction model developed based on the MRI features demonstrated excellent discriminatory performance.

## Introduction

Spinal myxopapillary ependymomas (MPEs) and schwannomas are intradural extramedullary tumors frequently arising around the conus medullaris and cauda equine^1^. In the recent 2021 World Health Organization (WHO) classification update of central nervous system tumors, MPE was regraded from grade 1 to grade 2, while schwannoma remained classified as grade 1^2,3^, which was caused by the partially aggressive clinical behavior exhibited by MPEs, which includes the possibility of disseminated disease, such as distant spinal or intracranial metastasis, and a relatively high rate of postsurgical relapse, occurring in up to 34% of cases^4–6^.

Appropriate surgical techniques for tumor removal are essential to treat MPEs, as they play a crucial role in determining outcomes^7^. Incomplete resection of the tumor and disruption of the tumor capsule are unfavorable prognostic markers, as they are associated with an increased likelihood of tumor recurrence and postoperative neurologic deficit^4,6,8,9^. Furthermore, the piecemeal removal technique, often used in minimally invasive approaches, may cause the tumor to spread through the cerebrospinal fluid^10,11^. Therefore, en bloc removal should be considered, whenever feasible, as the preferred approach for MPE. Contrastingly, spinal schwannomas exhibit slow progression and a low risk of recurrence despite incomplete tumor resection^12–14^. Consequently, in cases where postoperative neurologic deterioration is expected due to nerve root injury, subtotal removal of schwannomas may be indicated^13,14^. Hence, it is crucial to accurately differentiate between MPE and schwannomas before proceeding with surgical intervention.

MRI findings of spinal MPEs and schwannomas may exhibit similarities, potentially due to the presence of low cellular areas with abundant myxoid matrix^15,16^. However, published studies on MRI findings of spinal MPEs are mostly limited to case series or isolated case reports^16^. Moreover, there is a lack of comprehensive image analyses in studies comparing MRI findings between MPEs and schwannomas^17,18^. This scarcity of research could be attributed to the rarity of spinal MPEs and their recent recognition of potential aggressiveness, as evidenced by recent changes in their WHO tumor grade^2,3^.

This study’s primary goal was to retrospectively identify the significant MRI findings that can distinguish between spinal MPEs and schwannomas. Additionally, we aimed to develop a novel prediction model based on these findings that could play a vital role in guiding appropriate surgical approaches and predicting prognostic outcomes. Clinical parameters, such as symptoms at the time of diagnosis and recurrence during follow-up, were also compared.

## Results

### Patient and tumor characteristics

The comparison results of the clinical parameters between the MPE and schwannoma groups are summarized in Table 1. Patients with MPE showed a significantly higher frequency of bladder/bowel dysfunction (25% vs. 0%, *p* = 0.0006) and lower extremity weakness (33.33% vs. 3.77%, *p* = 0.0010) at initial presentation than those with schwannoma. Post-operative follow-up MRIs was performed in 15 and 51 patients with MPE and schwannomas, respectively. Although patients with MPE had significantly shorter follow-up period for imaging surveillance (mean ± standard deviation, 804.33 ± 801.62 vs. 1507.72 ± 617.12 days; *p* = 0.0009), two patients with MPE showed recurrence on follow-up MRIs and received postoperative radiation therapy, whereas patients with schwannoma showed had no recurrence (13.33% vs. 0%, *p* = 0.0490). The presence of bladder/bowel dysfunction and lower extremity weakness were chosen as clinical parameters for inclusion in Model 2.

**Table 1:** Comparison of clinical parameters between the myxopapillary ependymoma and schwannoma groups.

Parameters	Categories	Myxopapillary ependymoma (n = 24)^a^	Schwannoma (n = 53)^a^	*p*
Age (years)		44.21 ± 14.35	47.23 ± 13.6	0.4543^b^
Sex	Female	13 (54.17%)	28 (52.83%)	0.9133^c^
	Male	11 (45.83%)	25 (47.17%)	
Bladder/bowel dysfunction	Absent	18 (75%)	53 (100%)	0.0006^d^
	Present	6 (25%)	0 (0%)	
Lower extremity weakness	Absent	16 (66.67%)	51 (96.23%)	0.0010^d^
	Present	8 (33.33%)	2 (3.77%)	
Follow-up period (days)		804.33 ± 801.62	1507.72 ± 617.12	0.0009^b^
Recurrence^e^	Absent	13 (86.67%)	51 (100%)	0.0490^d^
	Present	2 (13.33%)	0 (0%)	
Lesion extent	Thoracic	2 (8.3%)	5 (9.4%)	0.2770^d^
	Thoracic-Lumbar	3 (12.5%)	4 (7.5%)	
	Lumbar	17 (70.8%)	42 (79.2%)	
	Lumbar-Sacral	0 (0%)	2 (3.8%)	
	Sacral	2 (8.3%)	0 (0%)	

^a^Data are expressed as mean ± standard deviation or number of patients with % within parentheses.

^b^indicates using Wilcoxon rank sum test.

^c^indicates using Chi-squared test.

^d^indicates using Fisher’s exact test.

^e^Data from 11 patients without follow-up imaging studies were excluded from this category (nine patients with myxopapillary ependymoma and two patients with schwannoma).

**Figure 1:**
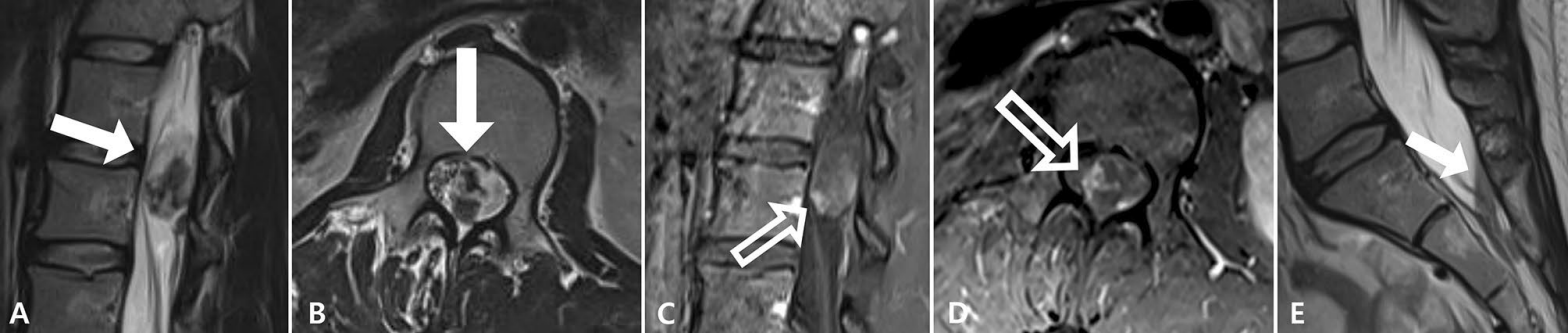
A myxopapillary ependymoma in a 25-year-old female patient at the L1-2 level. Sagittal (**A**) and axial (**B**) T2-weighted images show a 3.2 cm-sized intradural mass with heterogeneous signal intensity containing dark signal (white arrows). Sagittal (**C**) and axial (**D**) fat-suppressed contrast-enhanced T1-weighted images show focal enhancement pattern (open arrows). A fluid–fluid level is demonstrated in the distal dural sac, suggesting subarachnoid hemorrhage (**E**, black arrow).

**Figure 2:**
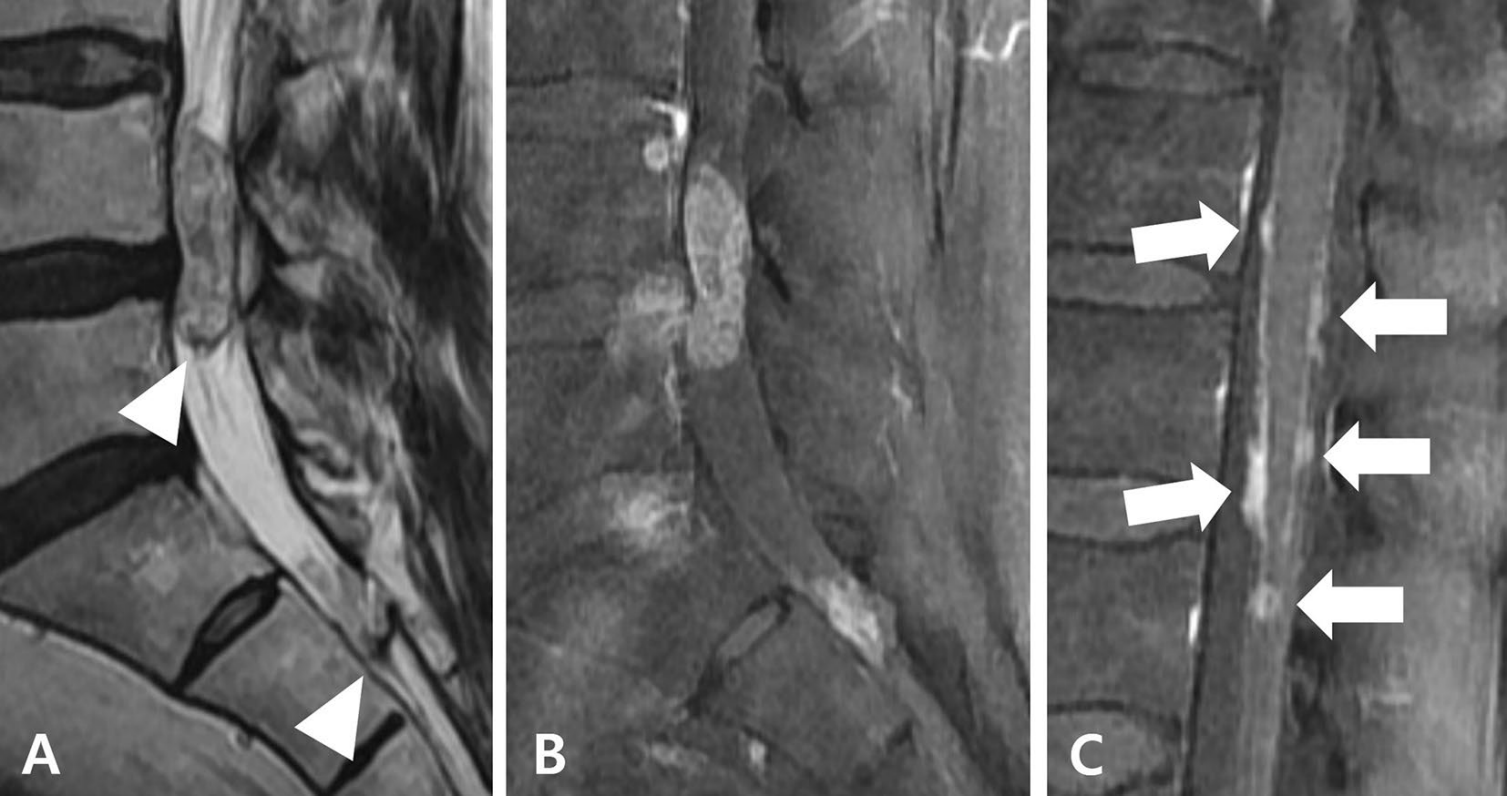
A myxopapillary ependymoma in a 65-year-old male patient at the L4-5 level. Sagittal T2-weighted (**A**) and fat-suppressed contrast-enhanced T1-weighted (**B**) images show a 4.2 cm-sized intradural mass at the L4-5 level with a leptomenigeal seeding at the distal dural sac. Both lesions demonstrate intratumoral T2 dark signal (arrowheads) and diffuse heterogeneous enhancement pattern. Sagittal fat-suppressed contrast-enhanced T1-weighted image at the thoracolumbar junction level (**c**) also shows multiple leptomeningeal seedings along the spinal cord surface.

**Figure 3:**
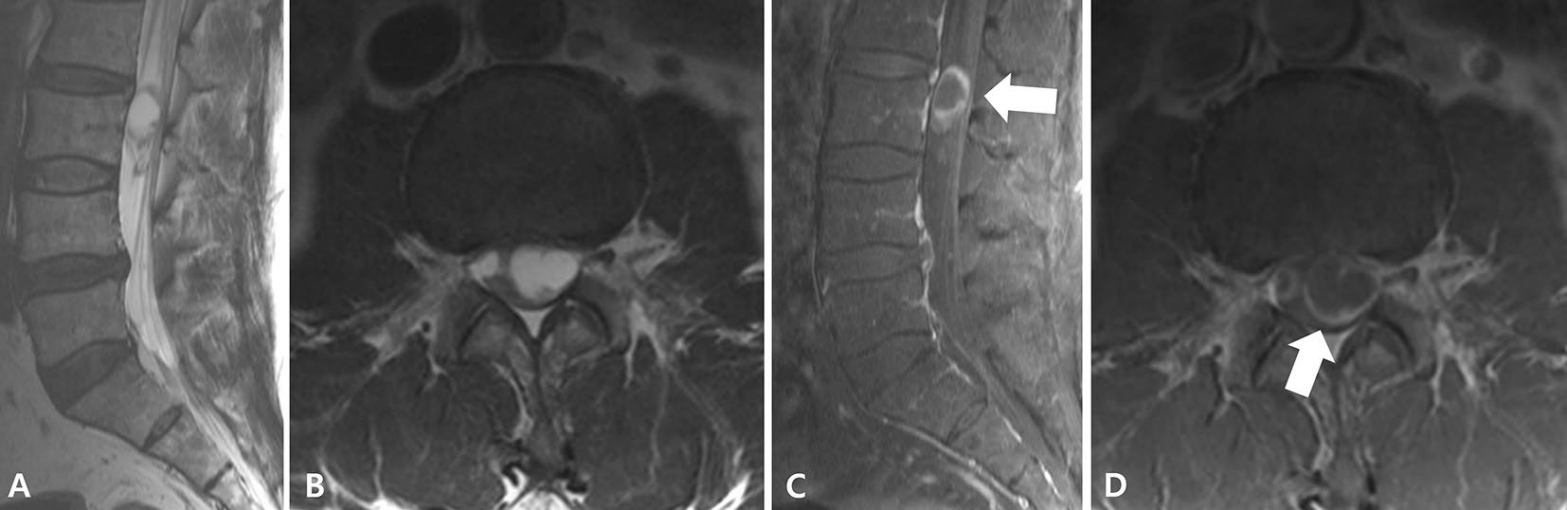
A schwannoma in a 59-year-old male patient at the L3 level. Sagittal (**A**) and axial (**B**) T2-weighted images show a 2.6 cm-sized intradural mass with hyperintense signal. Sagittal (**C**) and axial (**D**) fat-suppressed contrast-enhanced T1-weighted images demonstrate rim enhancement pattern (arrows) of the mass.

The univariable and multivariable analyses of the MRI parameters are summarized in Table 2. MRI analyses revealed that MPEs had significantly larger mean size (mean ± standard deviation, 37.93 ± 25.92 vs. 23.28 ± 9.98 mm; *p* = 0.0035). The optimal cutoff size for distinguishing MPE from schwannomas was determined to be 35 mm. Univariable analyses revealed that patients with MPE had a significantly higher frequency of tumor size equal to or larger than 35 mm (*p* = 0.0015), intratumoral T2 dark signal (*p* = 0.0021), associated spinal SAH (*p* = 0.0377), and leptomeningeal seeding (*p* = 0.0377) (Figs. 1 and 2). Using the rim enhancement pattern as a reference, most of which were observed in schwannomas (95.83%, 23/24) (Fig. 3), univariable analyses revealed that focal and diffuse heterogeneous enhancement patterns were significantly more common in MPEs (*p* = 0.0049 and 0.0038, respectively) (Figs. 1 and 2). Multivariable analyses showed that intratumoral T2 dark signal (*p* = 0.0439) and focal (*p* = 0.0029) and diffuse enhancement patterns (*p* = 0.0398) were independent factors. The *P* value from the Hosmer–Lemeshow test was 0.7087.

**Table 2:** Univariable and multivariable analyses of MRI parameters.

Variables	Categories	Total	Myxopapillary ependymoma	Schwannoma	Univariable analysis		Multivariable analysis	
					OR (95% CI)	*p*	OR (95% CI)	*p*
Size (mm)	< 35 mm^a^	60	13 (21.67)	47 (78.33)	ref	0.0015	ref	0.1787
	≥ 35 mm^a^	17	11(64.71)	6 (35.29)	6.63(2.06–21.34)		2.95 (0.61–14.25)	
Subarachnoid hemorrhage	Absent	72	19 (26.39)	53 (73.61)	ref	0.0377	ref	0.9207
	Present	5	5 (100)	0 (0)	30.19 (1.21–751.44)		1.24 (0.02–86.97)	
Leptomeningeal seeding	Absent	72	19 (26.39)	53 (73.61)	ref	0.0377	ref	0.0869
	Present	5	5 (100)	0 (0)	30.19 (1.21–751.44)		23.98 (0.63–910.9)	
Epicenter on axial image	Eccentric	31	7 (22.58)	24 (77.42)	ref	0.1854		
	Central	46	17 (36.96)	29 (63.04)	2.01 (0.72–5.65)			
Intratumoral fluid–fluid level	Absent	72	21 (29.17)	51 (70.83)	ref	0.1731		
	Present	5	3 (60)	2 (40)	3.64 (0.57–23.4)			
T2 dark signal	Absent	62	14 (22.58)	48 (77.42)	ref	0.0021	ref	0.0439
	Present	15	10 (66.67)	5 (33.33)	6.86 (2.01–23.4)		6.19 (1.05–36.42)	
Enhancement pattern	Focal	5	5 (100)	0 (0)	172.40 (4.75 to > 999)	0.0049	241.61 (6.25 to > 999)	0.0029
	Diffuse heterogenous	24	12 (50)	12 (50)	15.67 (2.43–101.14)	0.0038	8.89 (1.14–69.59)	0.0398
	Rim	24	1 (4.17)	23 (95.83)	ref		ref	ref
	Diffuse homogenous	23	5 (21.74)	18 (78.26)	4.66 (0.67–32.58)	0.1211	3.32 (0.39–28.16)	0.2312

Logistic regression analysis performed using Firth’s correction.

^a^The optimal cutoff size to distinguish myxopapillary ependymoma from schwannoma determined by maximizing Youden’s index.

OR, odds ratio; CI, confidence interval; ref, reference.

### Estimation of the prediction model

Based on the logistic regression, the equation for Model 1, which incorporated MRI parameters that demonstrated statistical significance in univariate analysis, was constructed using the following equation: The variables for focal, diffuse heterogeneous, rim, and diffuse homogeneous enhancements are represented by n, with values of 5.4873, 2.1853, 0, and 1.1991, respectively.

$$\left( A \right) y{ } = { }1.081{ }\left( {{\text{if}}\;{\text{size}} \ge 35\;{\text{mm}}} \right) + 0.2158{ }\left( {{\text{if}}\;{\text{SAH}}\;{\text{present}}} \right) + 3.1772{ }\left( {{\text{if}}\;{\text{leptomeningeal}}\;{\text{seeding}}\;{\text{present}}} \right) + 1.8227{ }\left( {{\text{if}}\;{\text{T}}2\;{\text{dark}}\;{\text{signal}}\;{\text{present}}} \right) + n{ }{-}{ }3.3279{ }$$

The equation for Model 2, which includes MRI and two clinical parameters – lower extremity weakness and bladder/bowel dysfunction, both of which demonstrated statistical significance – is as follows. In this equation, the values assigned to focal, diffuse heterogeneous, rim, and diffuse homogeneous enhancement are represented by “n”, specifically 5.4675, 1.5108, 0, and 0.8445, respectively:

$$\left( B \right) y{ } = { }2.0283{ }\left( {\text{if lower extremity weakness present}} \right) + 1.048\left( {{\text{if bladder}}/{\text{bowel dysfunction present}}} \right) + { }1.4979{ }\left( {{\text{if size}} \ge 35{\text{ mm}}} \right) + { }1.4622\left( {\text{if SAH present}} \right) + { }3.4258{ }\left( {\text{if leptomeningeal seeding present}} \right) + { }1.3318\left( {{\text{if T}}2{\text{ dark signal present}}} \right) + + { }n{ }{-}{ }3.3279$$

The probability ($p$) of the MPE for each patient can be calculated by inserting $y$ into Equation (C), providing a probability value between 0 and 1:

$$\left( C \right) p = \frac{{e^{y} }}{{1 + e^{y} }}$$

**Figure 4:**
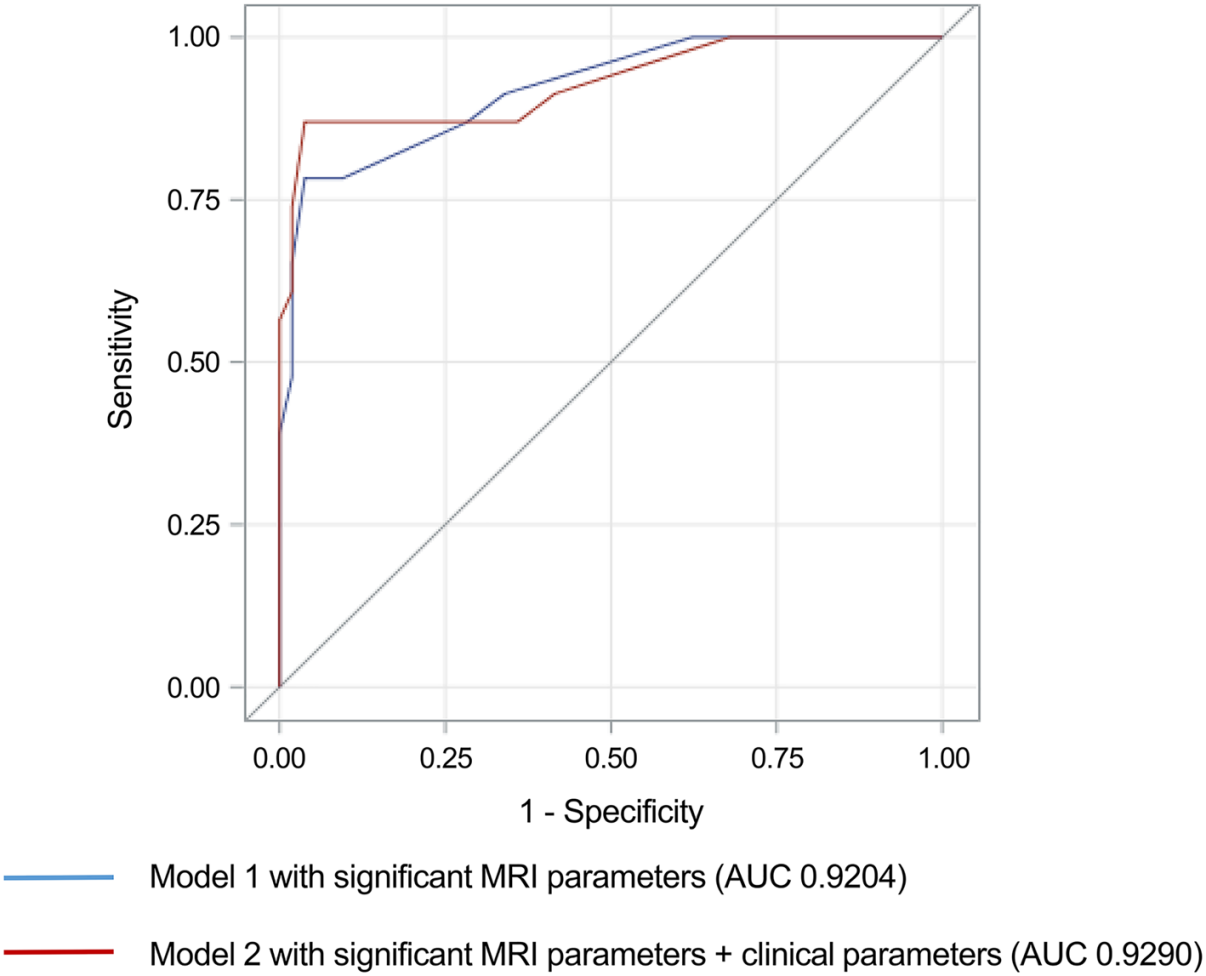
Receiver-operating characteristic curve analyses of the prediction models for differentiation of spinal myxopapillary ependymoma and schwannoma. The area under the curve (AUC) of Model 1, using magnetic resonance imaging (MRI) parameters that demonstrated statistical significance in univariable analysis, was 0.9204 (95% confidence intervals [CI], 0.8532–0.9876), while that of Model 2, developed by incorporating MRI and clinical parameters, was 0.9290 (95% CI 0.8567–1.0000). The difference in AUC between the two models was not statistically significant (*p* = 0.5426).

Model 1 showed an AUC of 0.9204 (95% confidence interval [CI], 0.8532–0.9876; Fig. 4). The average AUC for internal validation was 0.9210 (95% confidence interval, 0.9160–0.9270). With a predicted probability of MPE greater than 0.5, the sensitivity, specificity, positive predictive value, negative predictive value, and accuracy of the model were 0.652, 0.981, 0.938, 0.867, and 0.882, respectively. Model 2, which incorporated additional significant clinical parameters, yielded a slightly higher AUC of 0.9290 (95% CI 0.8567–1; Fig. 1) than Model 1. However, the difference in the AUC between the two models was not statistically significant (*p* = 0.5426).

Interobserver agreements were excellent for SAH (κ = 0.882; 95% CI 0.654–1.000), leptomeningeal seeding (κ = 1.000), intratumoral fluid–fluid level (κ = 0.882; 95% CI 0.654–1.000) and substantial for T2 dark signal (κ = 0.717; 95% CI 0.521–0.914), enhancement pattern (κ = 0.738; 95% CI 0.615–0.860), and location on axial image (κ = 0.695; 95% CI 0.544–0.846). The data obtained by one of the readers were used for comparison.

## Discussion

The objective of this study was to explore the MRI parameters that could potentially aid in the differentiation between spinal MPEs and schwannomas. Univariate analyses of MRI parameters showed that MPEs exhibited a significantly larger mean size as well as a significantly higher incidence of intratumoral T2 dark signal, focal and diffuse enhancement patterns, associated spinal SAH, and leptomeningeal seeding. Multivariate analyses revealed that intratumoral T2 dark signals and focal and diffuse enhancement patterns were independent factors discriminating between the two tumors. The constructed prediction model, utilizing MRI parameters that exhibited statistical significance in univariate analyses, demonstrated excellent diagnostic performance in discriminating between the two tumors. The results of the internal validation of the model also showed a similar discriminatory ability.

Published studies on MRI findings of spinal MPEs are mostly limited to case series or isolated case reports^6,16,19–22^. To the best of our knowledge, the largest case series of MPEs was reported by Wippold et al.^16^, which included the MRI findings in 20 patients with MPE. This study suggests that MRI findings related to MPEs lack specificity, except for a large, intensely enhanced intradural extramedullary tumor in the thoracolumbar spine. Two published studies have compared the MRI findings of MPEs and schwannomas^17,18^; however, these studies had several limitations from a methodological perspective in MRI evaluation without the involvement of a radiologist. Thus, we believe there is room for further investigation to identify MRI features that could help differentiate between MPEs and schwannomas.

Our results showed that MPEs had a significantly larger mean size and higher incidence of intratumoral T2 dark signals than schwannomas. A T2 dark signal within the MPE could be related to hemosiderin deposition due to hemorrhage, as the tumor is reported to be highly vascular and has a high risk of intratumoral bleeding^19,21,23^. This may explain the higher incidence of SAH associated with MPEs. However, the incidence of fluid–fluid levels within the tumor was not significantly different between the two groups. As there are other potential sources of decreased signal intensity on T2-weighted images, such as fibrocollagenous tissue or mineralization^24^, future studies with histopathological correlations of MRI findings are required to elucidate these findings.

Focal and diffuse heterogeneous enhancement patterns were significantly more commonly observed in MPEs, while the rim-enhancement pattern, which has been reported to be a major enhancement pattern in schwannomas, was also detected^25^. The difference in enhancement patterns between the two tumors may be attributed to differences in the cystic portion and vascularity in the two tumors. Histopathological cyst formation within the Antoni B region of schwannomas is linked to the rim-enhancement pattern demonstrated on contrast-enhanced MRI^26^. These results support the imaging sequences to differentiate between MPEs and schwannomas.

Patients with MPE showed a significantly higher frequency of bladder/bowel dysfunction and lower-extremity weakness at initial presentation. However, the reason for this remains unclear. One possible explanation could be that the relatively larger size and different histologic characteristics, including higher vascularity of MPE, might potentially cause higher pressure on the adjacent nerve structure, which could partially contribute to this clinical presentation. The different clinical presentations might be helpful in differentiating between the two tumors. However, the AUC of Model 2, which incorporated the two presenting symptoms alongside the MRI parameters, was higher than that of Model 1, albeit with a statistically insignificant difference. Further studies on the role of the clinical manifestations of MPE in its diagnosis are necessary.

In addition to the intrinsic limitations of this retrospective study, several other limitations exist. First, we could not perform external validation, which is essential for implementation in clinical practice. However, because of the rarity of MPE and because most of the literature consists of isolated case reports, we were unable to acquire additional datasets for external validation. Second, we lacked a histopathological correlation with the MRI findings of the tumor. Future studies with a histopathological correlation of MRI findings could be beneficial for confirming the true nature of MRI findings. Third, the clinical impact of preoperative tumor differentiation was not evaluated, but this was beyond the scope of our study. It would be interesting to evaluate the prospective clinical impact of preoperative tumor differentiation based on our prediction models in future studies. Fourth, a prediction model based on regression equations may have limited clinical utility. Developing a scoring system could be more useful and straightforward.

In conclusion, our results suggest that MRI can provide useful data to differentiate spinal MPEs from schwannomas. MRI features, including large size, presence of intratumoral T2 dark signal, focal and diffuse enhancement patterns, associated spinal SAH, and leptomeningeal seeding, were important discriminators suggesting the possibility of MPE. Clinical symptoms, such as bladder/bowel dysfunction and lower-extremity weakness, were more frequent in patients with MPE. Prediction models based on MRI and clinical features have demonstrated excellent discriminatory performances. Further investigations on the histological correlation and prospective clinical impact of preoperative differentiation are needed to validate our results.

## Materials and methods

### Study population

This retrospective study was approved by the Institutional Review Board (Samsung Medical Center Institutional Review Board, file no. 2023-04-132–001), which waived the requirement for informed consent. All methods in this study were performed in accordance with the relevant guidelines and regulations. We initially identified 363 consecutive patients who underwent surgical removal of spinal intradural extramedullary tumors at our institution and preoperative spine MRI between January 2012 and December 2022. Among these, we identified 24 patients with surgically confirmed spinal MPE. Because MPEs typically arise around the conus medullaris and filum terminale^27^, we chose patients with schwannomas located below the T11 upper endplate level because the cranial margin of the most cranially located MPE in our study population was T11 for comparison. We identified 112 surgically resected intradural extramedullary tumors located below the T11 upper endplate level on preoperative MRI, and 89 patients with schwannomas were detected among these. The excluded tumors included 12 meningiomas, three paragangliomas, two mature teratomas, one melanoma, one metastasis, one lymphoma, one angiolipoma, one lipoma, and one pilocytic astrocytoma. We additionally excluded 12 patients who had neural foraminal extension after preliminary MRI analysis by one radiologist (H.S.K, with 7 years of experience in musculoskeletal MRI), as such a case would not cause a diagnostic challenge for differentiation between the two tumors. Finally, 53 patients with schwannomas were included in the control group for comparison.

### MRI acquisition

MRIs were obtained using a 3-T system (Intera Achieva or Ingenia, Philips Medical Systems, Best, Netherlands). The standard MRI protocol comprised sagittal turbo spin-echo T1-weighted images (repetition time [TR]/echo time [TE], 422–875 ms/9–23 ms), sagittal and axial T2-weighted images (TR/TE, 3000–4500 ms/96–120 ms), and sagittal and axial T1-weighted images with fat suppression after intravenous administration of gadoterate meglumine (Gd-DOTA, Dotarem, Guerbet, Roissy CdG Cedex, France). Among the 77 preoperative spine MRI scans, all examinations except one MRI for a patient with spinal MPE were performed with contrast administration.

### Clinical and imaging parameter analysis

One radiologist (C.S) reviewed the electronic medical records, including age, sex, tumor histopathology, date of MRI examination, and surgery. The preoperative clinical assessment results were carefully reviewed, with special attention paid to lower extremity weakness and bladder/bowel dysfunction. For those with follow-up MRIs, the presence of recurrence and its management were investigated by reviewing medical reports.

Two board-certified radiologists (J.H.L and H.S.K, with 5 and 7 years of experience in musculoskeletal MRI, respectively) blinded to the histopathological diagnoses independently evaluated the MRI scans using picture archiving and communication system (Centricity Radiology RA 1000; GE Healthcare, Chicago, IL, USA). Each radiologist evaluated the presence of T2 dark signals and fluid levels within the tumors, spinal subarachnoid hemorrhage (SAH), and leptomeningeal seeding. A T2 dark signal was deemed present if a tumor contained an area with signal intensity similar to that of the ligamentum flavum, which may represent intratumoral hemosiderin deposition related to hemorrhage. Radiologists classified the enhancement patterns of the tumors as focal, rim, diffuse heterogeneous, or diffuse homogenous. Enhancement was categorized as focal when the enhancing portion accounted for less than half of the tumor. If the enhancing portion constituted more than half the tumor but not the entire area, it was classified as diffusely heterogeneous. Cases in which homogeneous enhancement was observed throughout the tumor were classified as diffusely homogeneous. When the enhancement was localized at the peripheral portion of the tumor exhibiting a rim appearance, it was classified as rim enhancement. Radiologists classified the epicenter of the tumor on axial images as central or eccentric. Cases in which an intradural mass caused the symmetrical displacement of the cauda equina were designated as having a central epicenter. Conversely, if the mass caused an asymmetrical displacement of the cauda equina, it was categorized as having an eccentric epicenter. Given the frequent origin of MPEs in the filum terminale, we hypothesized that the epicenter of the tumor on axial images could potentially aid in distinguishing them from schwannomas. One radiologist (H.S.K) recorded the tumor size, which was defined as the maximum of three orthogonal dimensions. The radiologist also recorded the extent of each tumor based on the corresponding spinal level where its cranial and caudal margins were located. For example, a tumor located at the T11-L1 level falls within the categorization of thoracolumbar.

### Statistical analysis

Continuous and categorical variables were summarized as means with standard deviations and frequencies (%). Logistic regression analysis was performed to assess the differences in MRI parameters between MPE and schwannomas for univariable and multivariable analyses using Firth’s correction. The goodness-of-fit was checked using the Hosmer–Lemeshow test. Clinical parameters were compared using the Wilcoxon rank-sum test, chi-squared test, or Fisher’s exact test. A prediction model (model 1) was developed using MRI parameters that demonstrated statistical significance in the univariable analysis. Furthermore, an additional model (Model 2) was developed, incorporating both MRI and clinical parameters that exhibited statistical significance. The area under the curve (AUC) was calculated based on receiver operating characteristic curve analysis of the prediction model, and the sensitivity, specificity, accuracy, and positive and negative predictive values were calculated. Among the variables included, the optimal cutoff value for size to distinguish MPE from schwannomas was determined by maximizing Youden’s index. We internally validated our prediction model using three repeated and stratified tenfold cross-validation techniques on the original dataset.

Kappa statistics were used to calculate the inter-observer agreement between the readers regarding SAH, intratumoral fluid–fluid level, T2 dark signal, enhancement pattern, and location on axial images. The degree of agreement was interpreted as ‘poor’ for a κ value of less than 0, ‘slight’ for a κ value of 0–0.20, ‘fair’ for a κ value of 0.21–0.40, ‘moderate’ for a κ value of 0.41–0.60, ‘substantial’ for a κ value of 0.61–0.80, and ‘excellent’ for a κ value of 0.81–1.0. All statistical analyses were performed using the Statistical Analysis Software (version 9.4, SAS Institute, Cary, NC, USA) and R 4.0.3 (Vienna, Austria; http://www.R-project.org). *P* < 0.05 was considered statistically significant.

## Data Availability

The datasets generated during and/or analyzed during the current study are available from the corresponding author on reasonable request.

## Abbreviations

MPE: Myxopapillary ependymoma
SAH: Subarachnoid hemorrhage
MRI: Magnetic resonance imaging
